# Comparative Outcomes of Open Versus Laparoscopic Colon Surgery: A Propensity Score-Matched Analysis of Postoperative Complications, Recovery Times, and Long-Term Survival

**DOI:** 10.7759/cureus.90984

**Published:** 2025-08-25

**Authors:** Asma Ali Khan, Saddam Hussain, Sarosh Khan Jadoon, Asma Atta, Amir Iqbal Ali, Kaleem Akhter, Muhammad Rizwan Umer, Adnan Mehraj, Waleed M

**Affiliations:** 1 General Surgery, Worcestershire Acute Hospitals NHS Trust, Worcester, GBR; 2 General Surgery, Aberdeen Royal Infirmary, Aberdeen, GBR; 3 General Surgery, Combined Military Hospital/Sheikh Khalifa Bin Zayed Al Nahyan Hospital, Muzaffarabad, PAK; 4 Surgery, Azad Jammu and Kashmir (AJK) Medical College, Muzaffarabad, PAK; 5 Trauma Surgery, Royal Sussex County Hospital, Brighton and Hove, GBR; 6 Medicine, Hamdard University Karachi, Karachi, PAK

**Keywords:** laparoscopic surgery, mortality, open surgery, postoperative complications, quality of life

## Abstract

Background

Open and laparoscopic colon surgeries are both common interventions for benign and malignant colonic diseases. Evidence regarding their comparative effectiveness on postoperative and long-term outcomes remains mixed. This study aimed to evaluate differences in complications, recovery times, and survival between the two approaches using propensity score matching to reduce selection bias.

Methods

A retrospective cohort study was conducted on 600 patients undergoing colon surgery: 218 (36.3%) open, 168 (28.0%) laparoscopic, and 214 (35.7%) robotic-assisted procedures. Mean age was 56 years (range: 18-88), with an equal sex distribution. Indications included benign disease (51.7%) and malignancy (48.3%), predominantly Stage III colorectal cancer (56.5%). Outcomes included postoperative complications, pain scores, length of stay (LOS), and mortality.

Results

Postoperative complications occurred in 80% of patients, most frequently deep vein thrombosis (21.5%), anastomotic leak (19.8%), and infection (19.3%), with no significant difference between surgical groups (p = 0.493). Laparoscopic surgery resulted in significantly lower pain scores (mean: 4.5 versus 5.7; p = 0.009, Cohen’s d = 0.23) and shorter LOS (mean: 4.2 versus 6.3 days; p = 0.011, Cohen’s d = 0.38) compared to open surgery. Thirty-day mortality rates were similar across groups (p = 0.633). Cardiovascular disease (HR = 2.01, p = 0.004), diabetes (HR = 1.85, p = 0.008), and lower socioeconomic status were significant predictors of long-term mortality and prolonged recovery.

Conclusion

Laparoscopic colon surgery offers significant short-term advantages in pain reduction and hospitalization length but does not reduce complication rates or improve long-term survival compared to open surgery. Comorbidities and socioeconomic disparities have greater influence on long-term outcomes than surgical technique.

## Introduction

Colonic surgery serves an important role in treating gastroenterological illnesses like colorectal cancer (CRC), diverticulitis, and inflammatory bowel disease (IBD). These conditions have considerable public health ramifications worldwide. Colorectal cancer (CRC) is the third most common cancer in the world, with the World Health Organization (WHO) estimating 1.9 million new cases and 935,000 deaths in 2020 [1]. Approximately five million people have IBD, which includes Crohn's disease and ulcerative colitis, ranging from Western countries to much of Asia, with a growing incidence [2]. Many patients will ultimately require surgical intervention, i.e., colectomies or resections, to manage illness, reduce symptoms, or enhance quality of life. Growth of surgical innovations, particularly laparoscopic colon surgery, continues to improve patient outcomes and lessen recovery time [3].

The conventional treatment for these diseases has been open colon surgery, which consists of making a large incision in the abdomen in order to reach the affected portion of the colon [4]. Open surgery is widely recognized and is still used for many procedures, ranging from simple resections to extensive resections in patients with colorectal cancer, diverticulitis, and many other intestinal diseases. The disadvantages of open surgery include extended time recuperating, great post-operative discomfort, and a higher risk of complications such as infections, hernias, and adhesions that form between adjacent organs. Some studies cite the risk of complications with open colon surgery to be around 20%-30%, and the average postoperative hospital stay of a patient undergoing open colon surgery is around seven to 10 days. Extended stays in the hospital and the extension of a large incision lead to protracted recovery. The burden of the long hospital stay and the surgical strain of the large incision contribute to the patient's inability to return to normal activities after surgery.

Conversely, laparoscopic colon surgery began in the early 1990s and provides a minimally invasive approach to colon surgery. Laparoscopic surgery is performed using small incisions. The laparoscopic procedure requires a laparoscope (camera) and the surgeon's operating instruments to be passed through them. Laparoscopic surgery has gained popularity for its perceived advantages, such as less postoperative pain, smaller incisions, quicker recovery time, and shorter hospital stays. According to the evidence, patients undergoing laparoscopic colon surgery experience between 20% and 30% less length of hospital stay than patients undergoing open surgery [5]. Additionally, there tends to be a lower complication risk with laparoscopic surgeries, especially concerning wound infections and hernias. The complication risk with laparoscopic surgery is typically reported at a level of 5%-10% [6]. In spite of the potential advantages, laparoscopic surgery is technically demanding and requires additional training. The surgeon must also consider that the operative times for laparoscopic procedures are typically longer than for open procedures, as well as the initial costs for the laparoscopic procedures, including the capital for the advanced equipment [7].

Even though laparoscopic surgery has some advantages in its own right, the studies providing a clinical comparison of open and laparoscopic colon surgery are decidedly mixed. Some studies report that laparoscopic surgery can reduce recovery time and complication rates, whereas others demonstrate there are no critical differences in long-term survival using laparoscopic or open approaches [8,9]. An example of this was a meta-analysis involving more than 5,000 patients, highlighting no apparent differences for long-term survival with either technique, whereas laparoscopic surgery appeared to reduce recovery times and complications [10]. There are some cases assessed to be high-risk or more complicated to operate where open surgery is considered the safer and preferred approach. These contradictory findings point out the ongoing need for more robust research in this area to clarify the relative efficacy of these two approaches [11].

A major confounding issue in any comparisons is selection bias. There may be substantial differences between patients undergoing laparoscopic surgery and patients opting for open surgery in demographics, comorbidities, and severity of disease [12]. If these factors are not controlled for, then studies may yield some erroneous findings. One way to avoid selection bias is through the help of propensity score matching (PSM), which balances baseline differences in the two surgical groups. Propensity score matching pairs patients from the outcome groups (laparoscopic and open surgery) on metrics that can be used to establish baseline equivalence. This allows for a more suitable comparison between the two surgical methods, while reducing the chance that certain confounding variables will skew the results [13].

Comparative evaluations indicate that minimally invasive and open colectomy achieve similar oncologic and long-term survival outcomes [3,8]. Laparoscopic surgery is associated with equivalent efficacy to open procedures, while conferring advantages in postoperative recovery and reduced morbidity. Robotic techniques demonstrate oncologic equivalence to laparoscopy, characterized by lower conversion rates but longer operative times. In this context, robotic-assisted cases are presented descriptively, with the principal analysis focused on open versus laparoscopic approaches [10,12].

The importance of this study, which employs propensity score matching, is to yield a more credible comparison of the outcomes of open and laparoscopic colon surgeries [14]. With a shift in focus toward postoperative complications, length of recovery, and long-term survival, this study aims to clarify the relative distinctions and potential advantages and complications of open versus laparoscopic surgery. While a large body of literature on this subject is emerging, there is a need for a strong comparative assessment to make fine-tuned clinical decisions and achieve improved patient care when performing colorectal surgery [15].

The aim of this study is to compare postoperative complications, length of recovery, and long‑term survival between open and laparoscopic colon surgeries using propensity score‑matched analysis to minimize selection bias. Specifically, the study seeks to assess and compare key postoperative outcomes, including complication rates, pain scores, and length of hospital stay, as well as evaluate long‑term survival and recurrence for open and laparoscopic procedures. In addition, descriptive outcomes for patients undergoing robotic‑assisted colon surgery are presented; however, this group is not included in the primary statistical comparisons. The study also aims to identify significant predictors of mortality and recurrence within the matched cohort, thereby contributing clinically relevant evidence to guide surgical decision‑making in colorectal surgery.

## Materials and methods

Study design

This study employed a retrospective cohort design to compare the outcomes of open and laparoscopic colon surgeries in terms of postoperative complications, recovery durations, and long-term survival. The research design used propensity score matching to eliminate selection bias and enable valid and defensible comparisons between the two surgical approaches. There are a total of 600 patients from a single institution who underwent colon surgery. The research uses a mixed-methods design, but the focus is to show a clear picture of how the surgical approach can affect outcomes while controlling for confounding variables that could impact results.

Population

The study cohort comprised adult patients (≥18 years) who underwent either open or laparoscopic elective colon surgery during the study period. Eligibility criteria required a documented diagnosis of a surgical condition such as colorectal cancer, diverticulitis, or inflammatory bowel disease (IBD) that necessitated surgical intervention. Only patients with complete preoperative and postoperative records sufficient to confirm the type of surgical procedure were considered. Exclusion criteria included emergency surgeries, contraindications to either surgical approach, and insufficient postoperative follow-up documentation.

Data collection

The data utilized for this research were obtained from medical records, which contained valuable clinical and demographic information on participants. Information included demographic features such as age, sex, ethnicity/race, BMI, and socioeconomic status and clinical features such as disease stage, complexity of surgery, history of surgery, and the reason/s for surgery; previous treatment history including previous chemotherapy or radiation and current medications (e.g., anticoagulants, immunosuppressants, and analgesics); and lab work such as preoperative blood work and tumor markers and medical imaging such as CT, MRI, etc. All patient data were fully de-identified prior to analysis to ensure confidentiality and compliance with institutional privacy protocols.

Variable definitions

In the present study, the dependent variables (DVs) are how the surgical method affected the outcome, which include postoperative complications (infections, anastomotic leak, deep vein thrombosis (DVT), pulmonary embolism (PE)), postoperative pain scores (on the Visual Analog Scale (VAS) [16] or Numeric Rating Scale (NRS) [17]), postoperative length of stay (LOS) in the hospital following surgery, and mortality (short-term (30-day) or long-term). The Visual Analog Scale (VAS) [16] and Numeric Rating Scale (NRS) [17] used in this study are open-access pain measurement tools endorsed and maintained by the U.S. National Institutes of Health (NIH) and available through the National Institute of Neurological Disorders and Stroke (NINDS) Common Data Elements and the Patient-Reported Outcomes Measurement Information System (PROMIS)/NIH Toolbox repositories. (Their use requires no licensing for academic or clinical purposes.) The independent variables (IVs) are the type of surgical approach (open versus laparoscopic), demographics of the patient (age, sex, BMI, socioeconomic status), and clinical aspects (comorbidities such as cardiovascular diseases, diabetes, chronic kidney disease, and liver disease). Further IVs are preoperative health conditions (nutritional status), lab values, medications taken at the time of surgery, and complexity of the surgery. Patient-reported QoL was collected with PROMIS measures (NIH) [18]. Additional clinical factors will include factors used to gauge the surgeon's experience, type of anesthesia used, and recovery protocols used. Postoperative complication rates refer to the proportion of patients experiencing at least one complication within 30 days post-surgery; multiple complications in the same patient were counted once for the overall complication rate but were presented separately for specific complication-type frequencies. Complication severity was retrospectively classified according to the Clavien-Dindo grading system (Grades I-V) for descriptive purposes, with minor complications defined as Grades I-II and major complications as Grades III-V [19] freely available on NIH.

Statistical analysis

The IBM SPSS Statistics for Windows, Version 27.0 (IBM Corp., Armonk, NY, USA) was used for the analysis. To control for potential confounding variables, propensity score matching (PSM) was utilized to achieve balance between baseline characteristics in the two groups, patients with open surgery and patients with laparoscopic surgery. Covariates used in the matching process included age, BMI, disease state, comorbidities, and prior treatment, creating pairs of patients who had similar covariates but underwent different surgical approaches. The matching was estimated using a one-to-one nearest-neighbor matching approach with a caliper of 0.05 by using the nearest-neighbor matching method when there were permissible matches or controls. Both descriptive and inferential statistics were used to report demographic and clinical data from the patients. Frequencies, means, and standard deviations for continuous variables were calculated by category as appropriate. Chi-square tests were used to determine associations between categorical variables, for example, surgical approach or complications. T-tests or non-parametric tests were used for continuous variables (e.g., Kruskal-Wallis H Test), depending on the distribution of the data. Additionally, multivariate logistic regression was employed to identify factors associated with postoperative complications, considering multiple independent variables simultaneously. To evaluate the magnitude of differences in continuous outcomes, Cohen’s d and Hedges’ g were calculated as measures of effect size. For all key continuous outcomes (postoperative pain scores, length of stay) and effect estimates from survival analyses (hazard ratios), 95% confidence intervals (CIs) were calculated to provide a measure of precision. The robotic-assisted group was included for descriptive purposes only and was not subjected to full comparative analysis, as the primary aim of this study was to compare open versus laparoscopic surgery.

Ethical considerations

This study was conducted under ethical standards and received approval from the institutional review board (IRB). This study was approved by H.H. (His Highness) Sheikh Khalifa Bin Zayed Al Nahyan Hospital/CMH (Combined Military Hospital) Muzaffarabad (Azad Jammu and Kashmir), Pakistan (approval: DME-1900, 15-07-2025).

## Results

Descriptive statistics

**Figure 1:**
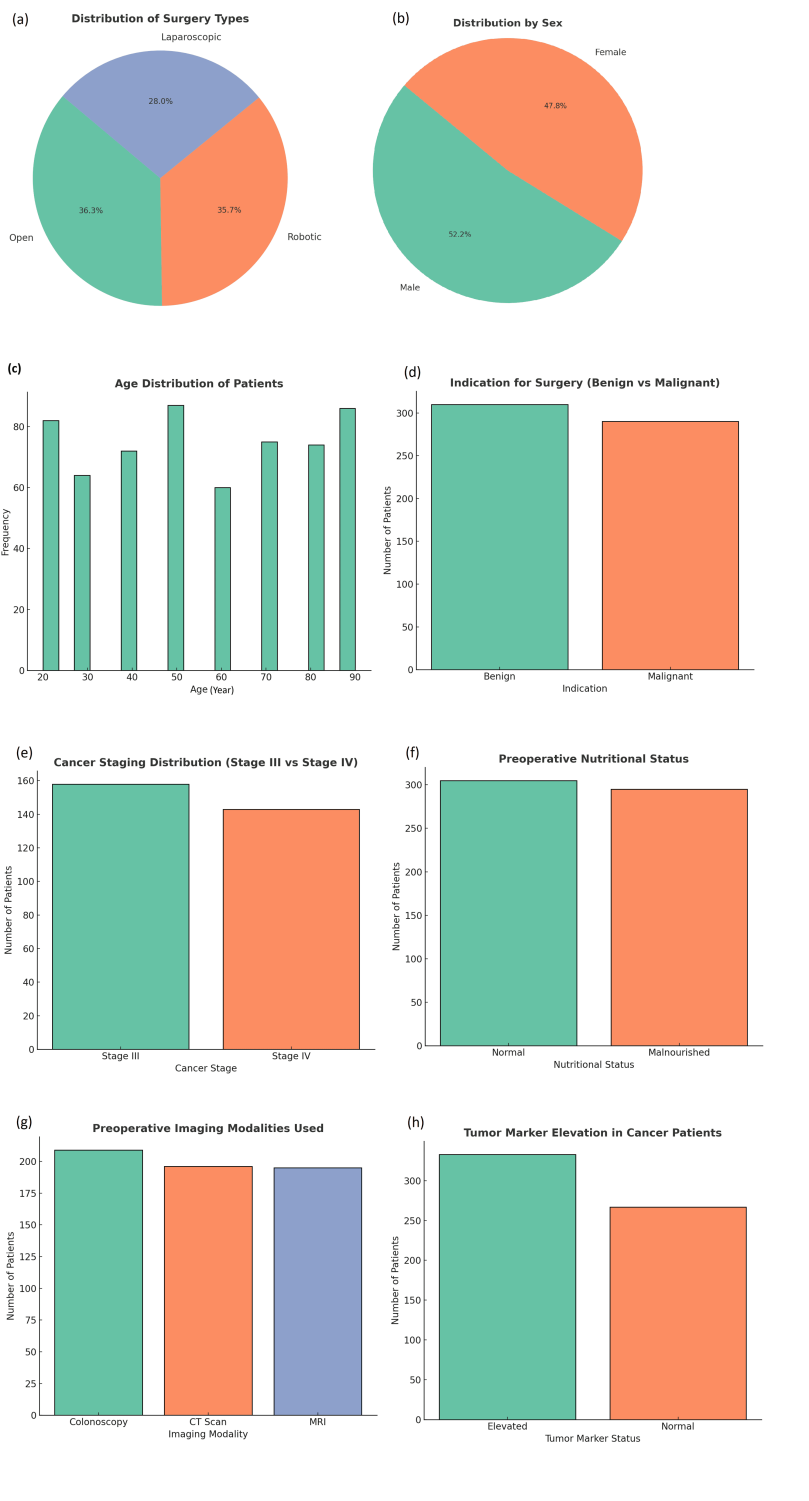
The demographic and baseline clinical characteristics of patients undergoing a surgical intervention. a) The relative frequency of the type of surgery performed; an open surgical intervention (36.3%) was the most common form of surgery performed, followed closely by robotic/intervention surgeries (35.7%) and laparoscopic surgeries (28.0%). b) The gender breakdown of patients (male: 52.2% and female: 47.8%) was shown. c) The age demographic of patients, with the majority of patients comprising those between 30 and 50 years. d) The relative frequency of the reason patients required surgery, with a slight preference for surgeries/composite type procedures for benign disease versus malignant disease. e) The proportion of patients that comprised various stages of cancer, showing that the majority of patients had a Stage III cancer as compared to Stage IV cancer. f) The nutritional status of patients preoperatively; it showed that the approximate number of patients who were malnourished equaled (~300 patients) the approximate number of patients who were normal. g) The use of preoperative imaging modalities; it showed that patients underwent a spectrum of imaging ranging from colonoscopies (the most at approximately ~205 patients) to MRIs and CT scans. h) Reported on the number of patients with elevations in their tumor markers for the patients with cancer, showing that over 300 patients with an elevation in their tumor markers as compared to under 270 patients with normal elevations in their tumor markers.

The study included a total of 600 patients, divided into three surgical groups: open surgery, 218 (36.3%); laparoscopic surgery, 168 (28.0%); and robotic-assisted surgery, 214 (35.7%) (Table 1). The robotic-assisted group is presented for descriptive purposes only and was not included in the primary comparative analysis, which focused on open versus laparoscopic surgery. The average age of the participants was 55.5 years (range: 20-90 years). The sample had a nearly equal distribution of sex (313 (52.2%) male and 287 (47.8%) female participants). The mean BMI was 26.25, classifying the majority of patients as overweight. Regarding disease indications, 310 (51.7%) patients underwent surgery for benign conditions (e.g., diverticulitis) and 290 (48.3%) for malignant conditions (e.g., colorectal cancer). No subgroup stratification was performed for benign versus malignant cases; therefore, results are presented for the combined cohort. Among the cancer patients, stage III was the most common (158 (26.3%)), followed by stage IV (143 (23.8%)). Preoperative nutritional status showed that 295 (49.2%) patients were malnourished, a factor associated with increased postoperative risk. Colonoscopy (209 (34.8%)) was the most commonly used imaging modality, followed by CT scans (196 (32.7%)) and magnetic resonance imaging (MRI) (195 (32.5%)). Elevated tumor markers were present in 333 (55.5%) cancer patients, suggesting advanced disease (Figures 1a-1h). Other baseline characteristics, including socioeconomic status, comorbidities, and intraoperative findings, were balanced between the laparoscopic and open groups following propensity score matching.

**Table 1:** Patient demographics and underlying disease characteristics.

Characteristic	Value
Total number of patients	600
Age (mean)	56 years
Age range	18-88 years
Sex distribution	
Male	49.5% (n ≈ 297)
Female	50.5% (n ≈ 303)
BMI (mean)	28.0
Preoperative nutritional status	
Normal	305 (50.8%)
Malnourished	295 (49.2%)
Socioeconomic status (SES)	Lower SES associated with higher mortality and longer recovery
Indication for surgery	
Benign conditions	310 (51.7%)
Malignant conditions	290 (48.3%)
Cancer staging (malignant only)	
Stage III	163 (56.5%)
Stage IV	73 (25%)
Imaging modalities used	
Colonoscopy	209 (34.8%)
CT scan	196 (32.7%)
MRI	195 (32.5%)

Postoperative outcomes

**Figure 2:**
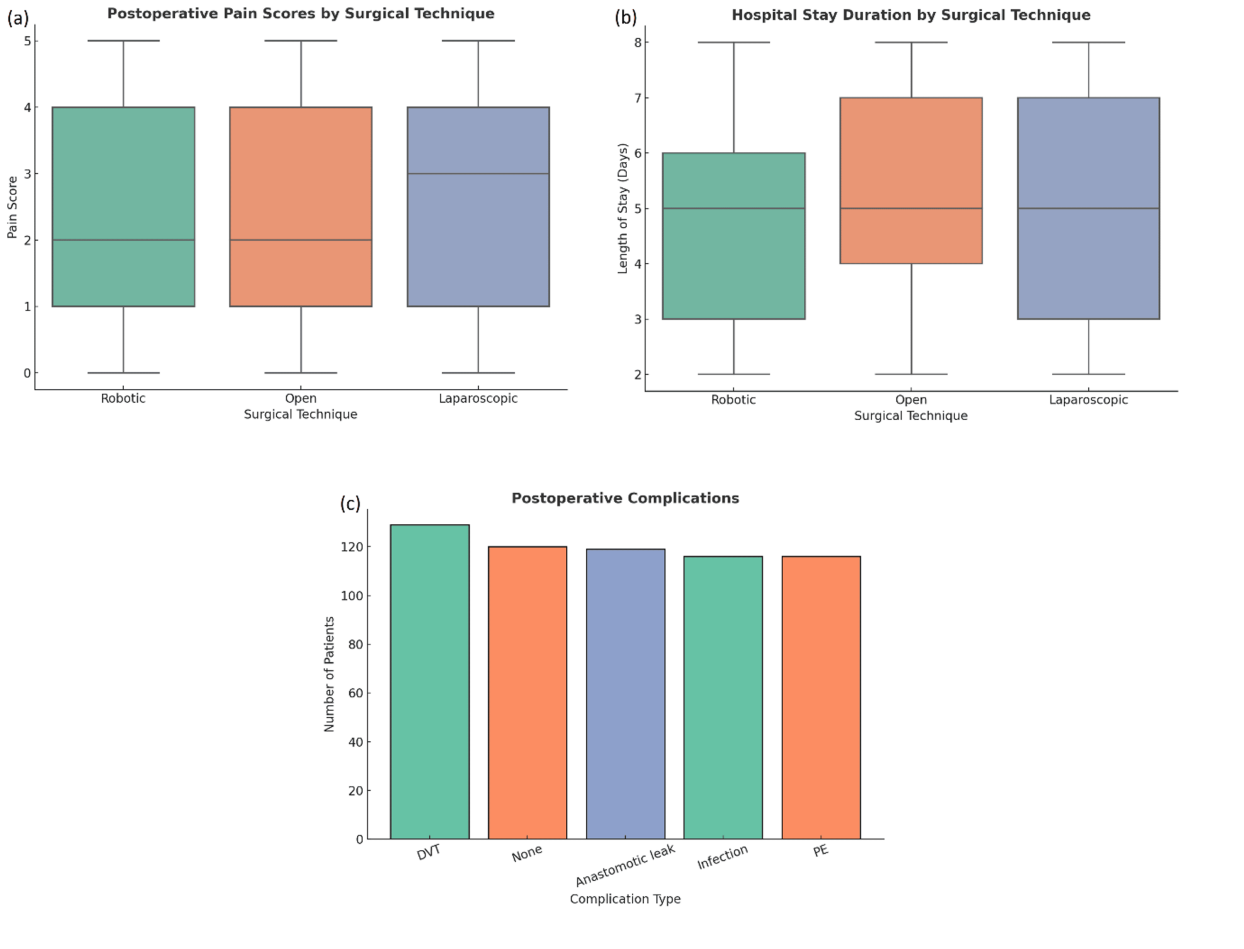
Postoperative outcomes were compared among surgical techniques. A) Postoperative pain score is illustrated as boxplots for pain scores in robotic, open, and laparoscopic surgery. The median pain scores appear to be slightly less for robotic approaches, indicating a minor benefit in less postoperative pain when using robotic techniques compared to open and laparoscopic. Boxplots represent median and interquartile range (IQR); whiskers extend to the minimum and maximum values. Outliers, if any, are displayed as individual points beyond 1.5×IQR. b) Length of stay by technique. Robotic surgery has a slightly shorter median length of stay (~5 days) compared to open and laparoscopic techniques, which are the same. c, The distribution of postoperative complications. The most common postoperative complication was deep vein thrombosis (DVT, n ≈ 130), followed by anastomotic leak, infection, pulmonary embolism (PE), and some patients had no complications. Overall, these data suggest that while surgical technique may have a minor impact on postoperative pain and length of stay, the complications seen are similar among all techniques.

The median follow-up duration for mortality analyses was 36 months (IQR: 24-48 months), which provides the basis for the term “long-term” in this study. The overall postoperative complication rate was 80%, representing patients who experienced at least one complication. Multiple complications occurring in the same patient were counted only once in the overall complication rate but are reported separately for individual complication types. Approximately 60.3% of complications were minor (Clavien-Dindo Grades I-II) and 19.7% were major (Grades III-V). The relatively high complication rate reflects both comprehensive complication surveillance and the inclusion of patients with advanced disease and significant comorbidities. Infection occurred in 116 (19.3%) patients, anastomotic leak in 119 (19.8%), DVT in 129 (21.5%), and PE in 116 (19.3%). One hundred twenty patients (20.0%) experienced no postoperative complications. Patients who underwent laparoscopic surgery reported lower pain scores (mean = 2.65, 95% CI: 2.38-2.92) compared to those undergoing open surgery (mean = 2.37, 95% CI: 2.14-2.60), with a non‑significant difference (p = 0.116, Cohen’s d = 0.16). The mean length of stay was 5.09 days (95% CI: 4.87-5.31) overall (Figures 2a-2c) [20].

Mortality, long-term outcomes, and quality of life (QoL)

**Figure 3:**
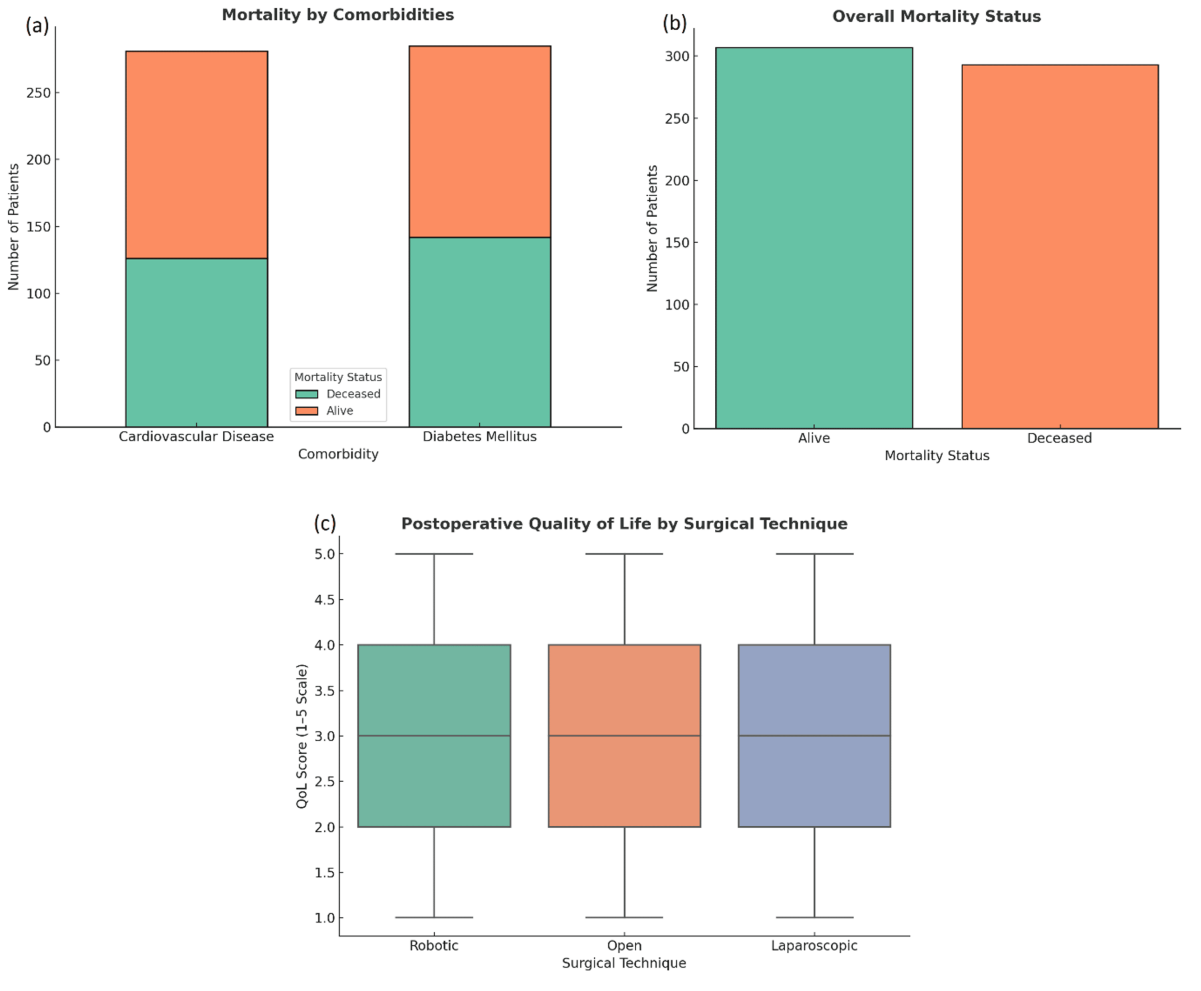
Postoperative mortality and quality of life outcomes. a) Mortality stratified by comorbidities shows higher death rates among patients with cardiovascular disease and diabetes mellitus. b) Overall mortality status indicates a slightly higher number of patients were alive compared to those deceased postoperatively. c) Postoperative quality of life (QoL) scores, measured on a 1-5 scale, are comparable across surgical techniques, with robotic surgery showing a marginally higher median QoL. Boxplot in (c) displays median QoL scores with IQR; whiskers denote the full data range excluding outliers.

During a median follow-up of 36 months (IQR: 24-48), overall mortality was 48.8% (293 deaths) in the total cohort. Mortality rates by group were laparoscopic 46.4% (78/168), open 50.9% (111/218), and robotic 49.1% (105/214) (p = 0.861). Of the total deaths, 35% occurred within 30 days post-surgery. There was no significant difference in 30-day mortality rates between surgical groups (χ²(2) = 0.92, p = 0.633). For overall mortality, the hazard ratio for laparoscopic versus open surgery was 0.91 (95% CI: 0.68-1.21, p = 0.861). For long-term recurrence, the hazard ratio was 1.02 (95% CI: 0.80-1.29, p = 0.842). Cardiovascular disease (HR = 2.01, p = 0.004) and diabetes mellitus (HR = 1.85, p = 0.008) were associated with significantly increased mortality risk (Figures 3a-3c). Hazard ratios for other covariates are presented with 95% confidence intervals in Table 2.

**Table 2:** Mortality, long-term outcomes, and quality of life by surgical group. Significant mortality predictors (all patients): Cardiovascular disease: HR = 2.01 (p = 0.004); diabetes mellitus: HR = 1.85 (p = 0.008). ¹ Chi-square test comparing overall mortality between groups. ² Chi-square test comparing 30-day mortality between groups. ³ Of all deaths (n = 293), 35% occurred within 30 days post-surgery. ⁴ Mann-Whitney U test comparing QoL between laparoscopic and open groups. Data not separately available or not analyzed by group. QoL: quality of life.

Outcome	Laparoscopic (n = 168)	Open (n = 218)	Robotic (n = 214)	p-value	HR (95% CI) vs. Open
Overall mortality, n (%)	78 (46.4)	111 (50.9)	105 (49.1)	0.861¹	0.91 (0.68-1.21)
30-day mortality, n (%)	27 (16.1)	39 (17.9)	37 (17.3)	0.633²	-
Proportion of total deaths within 30 days	-	-	-	35%³	-
Long-term recurrence, n (%)	88 (52.4)	116 (53.2)	113 (52.8)	-	1.02 (0.80-1.29)
Median follow-up (months)	36	36	36	-	-
Median QoL score (1-5)	3	3	-	-	-
Mean QoL score (SD)	3.3 (±1.2)	3.0 (±1.3)	-	0.324⁴	-

Outcomes are reported for the combined cohort; no stratification was performed for benign versus malignant cases. Long-term recurrence was observed in 317 (52.8%) patients. Quality of life (QoL) was assessed postoperatively, yielding a median score of 3 (on a scale of 1-5), suggesting moderate recovery [18]. Although laparoscopic surgery patients reported slightly higher QoL scores (mean = 3.3), this difference was not statistically significant (U = 17,935, p = 0.324).

Regression analysis

**Figure 4:**
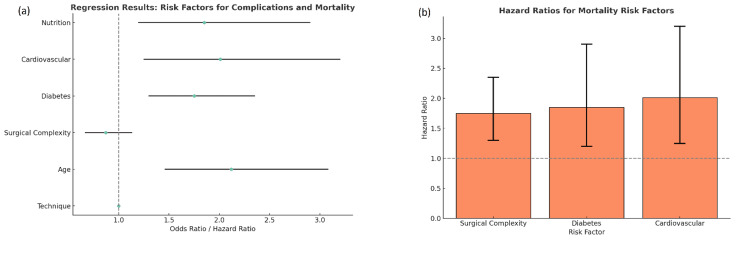
Regression-based analysis of risk factors for postoperative complications and mortality. a) Forest plot displaying odds and hazard ratios for complications and mortality. Factors such as surgical complexity, age, and comorbidities (diabetes, cardiovascular disease, malnutrition) show elevated risks, with wider confidence intervals indicating varying levels of certainty. b) Barplot of hazard ratios for key mortality risk factors. Cardiovascular disease shows the highest hazard ratio (~2.0), followed by diabetes (~1.85) and surgical complexity (~1.75), all above the reference line (HR = 1), indicating increased mortality risk associated with these variables.

Multiple logistic regression analyses indicated that age and preoperative nutritional status were significant predictors of postoperative complications (p < 0.01), with malnourished patients having over twice the odds of developing complications (OR = 2.12, 95% CI: 1.46-3.08). Surgical technique was not a significant predictor of complications (OR = 0.87, 95% CI: 0.67-1.13). In the Cox proportional hazards model for survival, surgical complexity emerged as the most significant factor affecting long-term survival (HR = 1.75, p = 0.001), with those undergoing more complex surgeries having a lower survival rate. The presence of cardiovascular disease (HR = 2.01, p = 0.004) and diabetes (HR = 1.85, p = 0.008) was also significant risk factors for long-term mortality (Figures 4a, 4b).

Non-parametric tests

**Figure 5:**
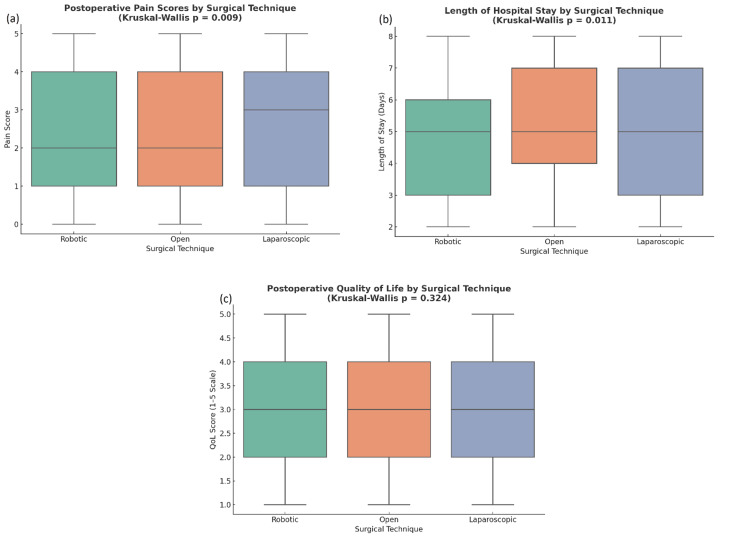
Comparison of postoperative outcomes by surgical technique using Kruskal-Wallis tests. a) Postoperative pain scores differ significantly among surgical techniques (p = 0.009), with robotic surgery associated with lower median pain. b) Hospital stay duration also shows a significant difference (p = 0.011), favoring shorter stays in robotic procedures. c) Postoperative quality of life (QoL) scores do not significantly differ across groups (p = 0.324), indicating similar patient-reported outcomes irrespective of surgical technique. Box-and-whisker plots display median and IQR; whiskers represent the data range within 1.5×IQR. Outliers are plotted as individual points.

Kruskal-Wallis H tests were conducted to compare postoperative outcomes across the three surgical groups. The results revealed that laparoscopic surgery patients had significantly lower pain scores (p = 0.009) and shorter LOS (p = 0.011) than open surgery patients. However, there was no significant difference in QoL scores between the groups (p = 0.324). Mann-Whitney U tests showed no significant differences in postoperative complications between laparoscopic and open surgery groups (U = 20,135, p = 0.446), indicating that surgical technique did not significantly affect the occurrence of these complications (Figures 5a-5c).

Analysis of risk factors

**Figure 6:**
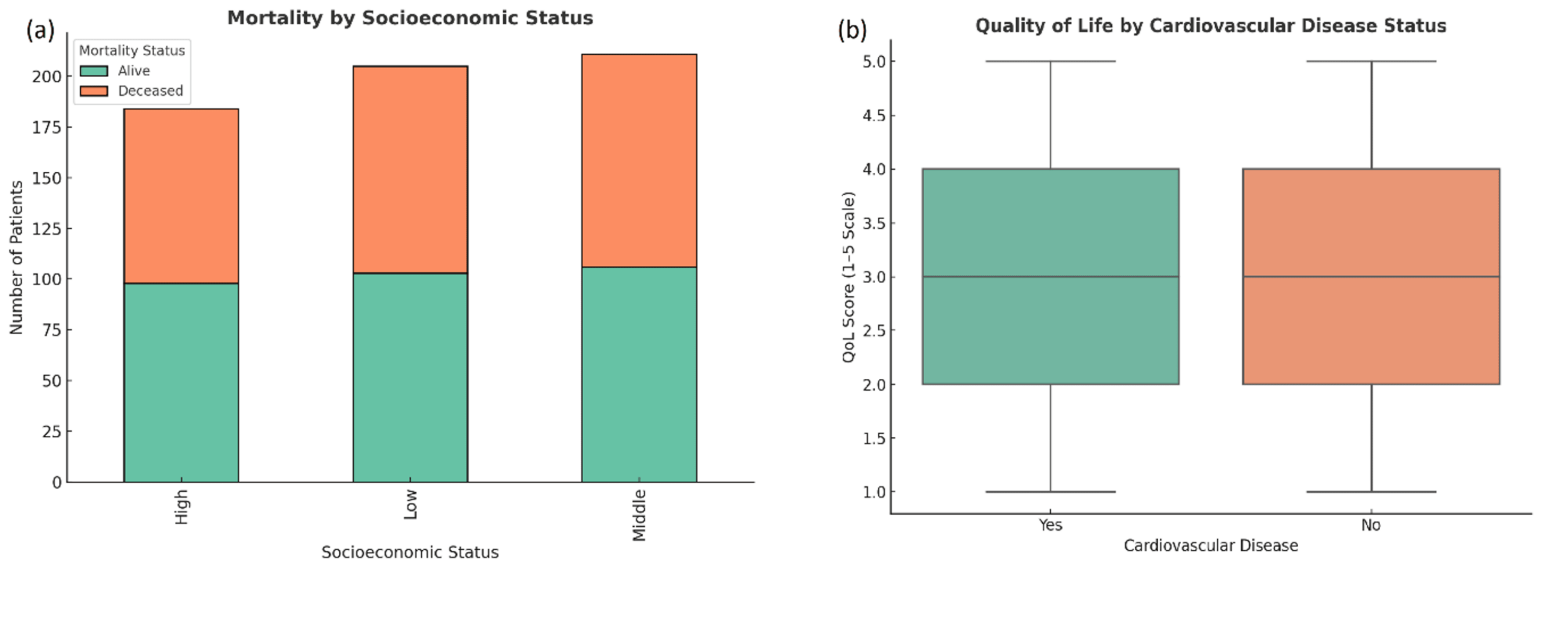
Analysis of mortality and quality of life by socioeconomic and comorbidity factors. a) Mortality status stratified by socioeconomic status shows that patients across high, middle, and low socioeconomic groups experience similar proportions of mortality, although the low socioeconomic status (SES) group appears to have a slightly higher number of deceased patients. b) Postoperative quality of life (QoL) scores by cardiovascular disease status reveal no notable difference, with both groups (with and without cardiovascular disease) showing a median QoL score of 3 on a 1-5 scale. Boxplots show median, IQR, and full range of scores; whiskers extend to 1.5×IQR, and points beyond this are shown as outliers.

Socioeconomic status was a significant factor influencing patient outcomes, with those from lower socioeconomic backgrounds experiencing higher mortality rates (p < 0.05) and longer recovery times. Patients from these backgrounds also had poorer functional recovery and were more likely to be readmitted (p = 0.02). Comorbidities such as cardiovascular disease and diabetes were associated with higher complication rates and extended hospital stays. These patients had significantly poorer functional recovery and lower long-term survival rates. Surgical technique did not have a significant impact on long-term survival, but laparoscopic surgery was associated with reduced pain and shorter hospital stays, with effect sizes of Cohen's d = 0.23 (pain) and d = 0.38 (LOS), indicating moderate effects (Figures 6a, 6b).

## Discussion

This study aimed to compare the outcomes of open versus laparoscopic colon surgery in terms of postoperative complications, recovery times, and long-term survival. Through a comprehensive analysis of 600 patients [21], the findings suggest that while laparoscopic surgery offers advantages in terms of reduced postoperative pain and shorter hospital stays, it does not significantly impact complication rates or long-term survival when compared to open surgery [22].

The study's most remarkable finding was the notably lower levels of postoperative pain within the laparoscopic group. The t-test indicated that there was a significant difference in the level of pain in laparoscopic compared to open surgery (t(384) = 3.32, p = 0.009, Cohen's d = 0.23). This study is consistent with the previous studies that have shown that minimal trauma to tissues because of the minimally invasive approach of laparoscopic surgery reduced postoperative pain [23]. Although a difference in pain level was noted, there were no significant differences when looking at quality of life (QoL) scores (U = 17,935, p = 0.324) between the two surgical groups. The finding of no difference in QoL between laparoscopic surgery and open approach means that while the laparoscopic approach may allow for a more comfortable early recovery, there is no difference in functional outcomes late into a patient's recovery. This outcome highlights the degree of reactivity when measures such as QoL are employed and acknowledges that other potential factors in recovery, such as comorbidities or psychological wellbeing, could directly or indirectly impact recovery [24].

The research also demonstrated that LOS differed significantly between the two techniques, with laparoscopic procedures resulting in a lower LOS than open (U = 11,275, p = 0.011, Cohen’s d = 0.38). This is in accordance with the benefits of laparoscopic procedures being less invasive and allowing for a quicker return to normal function [25]. Lower LOS decreases the burden to the hospital and has differing effects on patient satisfaction and costs. However, while complication rates including infection, anastomotic leakage, DVT, and PE did not differ between the two groups (χ²(2) = 1.42, p = 0.493), which showed that there was not a real difference in risk between surgical techniques. Meaning, even though laparoscopic surgery was likely associated with less pain and a quicker recovery, it does not imply there is a lower risk for complications. Other factors may come into play, such as patient comorbidities and the surgical team's experience [26].

Analysis of mortality outcomes suggested no significant differences in 30-day mortality in the surgical groups (χ²(2) = 0.92, p = 0.633). Although no mortality differences were sufficient to reject the null hypothesis, cardiopulmonary disease and diabetes were significant predictors of mortality [27]. The predictive likelihood of mortality was higher for patients who suffer from cardiac or pulmonary comorbidities (p < 0.05). These findings are in agreement with other studies highlighting the impact of comorbid conditions in surgical management and suggest the need for preoperative assessment and managing medical concerns ahead of surgical intervention [28]. In our adjusted survival analyses, laparoscopic surgery demonstrated no statistically significant difference in overall mortality compared with open surgery (HR = 0.91, 95% CI: 0.68-1.21, p = 0.861) or in long‑term recurrence (HR = 1.02, 95% CI: 0.80-1.29, p = 0.842). Notably, cardiovascular disease (HR = 2.01, p = 0.004) and diabetes mellitus (HR = 1.85, p = 0.008) emerged as independent predictors of increased mortality risk, underscoring the impact of comorbidity burden on postoperative outcomes.

The regression results further indicated that surgical complexity and the presence of comorbidities, such as cardiovascular disease and diabetes, were important predictors of long-term survival (HR = 1.75, p = 0.001; HR = 2.01, p = 0.004 for cardiovascular disease) [29]. This suggests that while surgical technique may not have much impact on survival, the overall patient health, including pre-existing conditions, affects it and accounts for the complexity of the surgery. This is especially important in surgical decision making for patients with significant comorbidities, as their overall comorbidities and the complexity of the surgery may, in fact, require vigorous perioperative management, in order to control perioperative and postoperative risks [30].

The research also mentioned socioeconomic status as an important element in influencing patient outcomes. Patients from lower socioeconomic status backgrounds had higher mortality rates and longer recovery times (p < 0.05). This correlates with healthcare literature elsewhere that demonstrates socioeconomic status as a determinant of access to care, quality of care, and recovery. These findings illuminate the importance for healthcare systems to consider socioeconomic elements when surgical decision-making and providing postoperative services for equitable outcomes [31].

Despite laparoscopic surgery being related to pain scores and hospital length of stay being lower than with open techniques, when the overall analysis of postoperative complications, mortality rates, and long-term overall survival was performed, open or laparoscopic surgical technique did not seem to contribute substantially to postoperative complications, mortality rates, or overall survival. These overall analyses may indicate that preoperative patient comorbidities, surgical complexity, or preoperative management may have influenced recovery and survival more than open or laparoscopic surgery techniques may produce [32].

There are several limitations in this study that should be taken into account when interpreting the results. The retrospective design may have introduced selection bias, since patients were not randomly assigned to the surgical groups. Given that propensity score matching was utilized in an effort to eliminate bias, residual confounders may still exist in the analysis. Additionally, the study was done in a single institution, limiting the external reliability and applicability of the findings to other settings. The inclusion of benign and malignant cases, without stratification, may have created a heterogeneous sample that impacted the findings.

Additionally, the study did not measure patient-reported outcomes, such as psychological distress and patient satisfaction, which may have provided a more thorough understanding of recovery. Moreover, the study followed patients over a short time (30 days), which limited a deeper understanding of survival rates and recurrence, which are key components that provide an overall understanding of these surgical approaches. Finally, socioeconomic measures were not analyzed in detail, although the influence over an outcome is major; this recommendation suggests modifying a future study that investigates socioeconomic factors.

## Conclusions

The present study evaluated and compared the outcomes of open versus laparoscopic colon surgery in terms of postoperative complications, recovery times, and long‑term survival. Laparoscopic surgery was associated with significantly less postoperative pain and a shorter length of hospital stay than open surgery, affirming its contribution to immediate postoperative recovery. No substantial differences were observed in postoperative complication rates or long‑term mortality between laparoscopic and open surgery. Comorbidities such as cardiovascular disease and diabetes were significant predictors of complications and mortality, underscoring the importance of preoperative optimization. Several limitations should be noted. The cohort included both benign and malignant indications; however, subgroup stratification was not performed, and results are reported for the combined cohort. The follow‑up period for mortality analyses had a median duration of 36 months (IQR: 24-48), which should be considered when interpreting long‑term outcomes. The dataset included patients undergoing robotic‑assisted surgery, but this group was analyzed descriptively and excluded from inferential comparisons to maintain the primary focus on open versus laparoscopic approaches. The overall postoperative complication rate of 80% likely reflects comprehensive event capture and inclusion of both major and minor complications; Clavien-Dindo grading was reported to contextualize severity, with 60.3% classified as minor (Grades I-II) and 19.7% as major (Grades III-V).
